# Professor Pancoasts Clinic, (Surgical)

**Published:** 1844-11-30

**Authors:** Edward R. Squibb


### PROFESSOR PANCOASTS CLINIC, (SURGICAL.)

Reported by Edward R. Squibb.

*Saturday, Nov.* 9, 1844.

The Lecturer commenced by saying that it was not customary for him in that place, to occupy much time in remarks prefatory to his course of Clinical Lectures. He restricted himself, therefore, to a few observations in reference to the arrangements made by the Managers of the Institution, for the purpose of diffusing as widely as possible, the facilities of the house for clinical instruction,—alluded to the spacious surgical wards of the house, and the great number of patients they contained, which yielded opportunities during the season for the illustration of a great variety of surgical affections. It was probable that in the course of the winter the class would have an opportunity of witnessing many operations. But, he stated, that it would be his particular object to attract attention to that which was less well described in books, namely, the diagnosis, pathology and treatment of surgical diseases; and should the treatment fail to effect a cure, the operations would then be considered, which as a last resort are rendered necessary, either to save the life, or diminish the sufferings of the patient, or facilitate the progress of cure that would otherwise be protracted.

The operation of Lithotomy, which he had expected to perform to day, had been necessarily postponed in consequence of the suffering of the patient from a severe fit of stone, attended with a suppurative discharge from the bladder; but he remarked that if the patient improved sufficiently, he would probably bring him forward on a subsequent occasion.

The first case to which the attention of the class was directed, was one of a very interesting character, from its nature, as well as from the many difficulties which it presented to successful treatment. It was that of a young gentleman from Tennessee, in whom the excessive use of mercury, fourteen years ago, had been followed by extensive destruction of the right cheek, lip, and ala of the nose; one of the consequences of the healing of the ulcer had been the formation of a band of cicatrix between the jaws, as large in diameter as the tendo-achillis, fastening them immovably together, and constituting a false anchylosis.

This having occurred prior to the period of second dentition, the permanent teeth when they appeared, grew very irregularly; some of them projecting through the opening in the cheek, owing to the obstruction presented to their natural course, leaving fissures through which alone, the patient had been able to take his food for 13 years.

The indications to be fulfilled in such a case are three in number; namely, to render the jaw movable, to replace the soft parts of the cheek and lip, and to restorè the side of the nose; the two last of which must be fulfilled by plastic operations: to accomplish these objects successfully will require some time, as each is to be attained by a separate process, that should be finished before the next is attempted.

The first operation, or that for the cure of the anchylosis, is one always modified by the nature of the obstacle to be overcome. If the anchylosis was true, or consisted in a deposition of bone in and around the joint, no operation of the kind which I propose here would be available or proper. In such a case nothing but the establishment of a false joint as proposed by Dr. Barton of this city, by sawing through the bone below its condyles, and keeping up a motion in the parts during the process of healing, could be attempted with any probability of success, and this, the result of which would necessarily be doubtful, has not yet been practised upon the living subject. On the other hand—and hence the necessity of a correct diagnosis,—when the anchylosis is false, or does not consist of a bony deposit, the joint remaining with all its structures for motion perfect, as evinced by the scarcely perceptible motion which we here give to the jaw, the means of relief are much less difficult, and to be undertaken with greater chance of success.

The resistance to be overcome in the case before us, is made by what was technically called by Delpech *inodular tissue*, the result of the organization of effused lymph on the inner suppurating surface of the ulcer in the cheek, left by the detachment of the slough. This lymph undergoing gradual condensation during the healing process, has passed through the phases of cellular and fibrous tissue, and in this instance presents to the touch a feeling of hardness almost like that of bone. The band is here short, but very thick and strong, being attached by its extremities to the outside of the gums of the upper and lower jaws, and by its outer surface to the remaining fragment of the cheek. In operating upon such cases, simple division of the band of cicatrix is not found sufficient to effect any lasting benefit, for union is quickly re-established in despite of every effort to prevent it, and the parts return to their former condition. The whole of this inodular tissue should therefore be removed by excision; to do which, however, I have never found it necessary,—as recommended, and practiced by Dr. Mott, of New York, in one case—to lay open the cheek from the corner of the mouth to the ramus of the jaw, in order to render the band accessible, allowing the edges of the wound in the cheek to cicatrize until the play of the jaw was restored, and then excising the edges and closing them as in the hair lip operation. In every case as yet, I have been able by drawing back the corner of the mouth, to divide the band by two or three cross cuts, and extirpate it in separate portions. In the case before us, in consequence of the loss of structure in the cheek, the extirpation is render more easy than usual.

The fixed condition of these parts for thirteen or fourteen years will not admit of our supposing the patient capable of opening his mouth immediately on the divison of the band, for the muscles, viz. the masseter and temporal, after remaining in a state of rest for so long a time lose their disposition to relaxation, and become fixed in their shortened state, like the firmly retracted muscles in club foot; hence the necessity will arise after the section of the cicatrix of using some mechanical means whereby the jaws may be separated and these muscles extended and placed in the condition necessary for the exercise of their functions. For this purpose the old jaw dilator of Heister, consisting of two hinged plates, which are forcibly separated by means of a screw, answers very well. In case these means should fail in separating the jaws to a sufficient extent it may become necessary to divide one or both masseter muscles. The patient, who is somewhat interested in the progress of medical and chirurgical science, has with some philosophy made up his mind to have the operations exhibited to the class, and by this he places it under some obligation.

*Operation.*—(The first step of the operation was then performed, viz., the separation of the skin from the cicatrix, then a separation of the cicatrix from the bones, thirdly, the removal of the cicatrix which was found exceedingly firm and hard.) After this removal of the band the jaw is found to yield somewhat, but upon further inspection the gums, where the double teeth should be, are found adherent, and it becomes necessary to dissect them apart. The anterior part of the right masseter muscle also appears so much contracted as to require division, which I shall now make from before backwards with the bistoury. I now apply the dilator, and by its means the jaws are separated without much difficulty; by extending its application to the other side, still more space is gained.

The jaws are now opened to the extent of an inch, and with this we shall have accomplished sufficient for the day. We shall direct the daily application of the instrument in order gradually to increase the separation to the natural extent; keeping during the intervals pieces of cork interposed between the teeth, in order to preserve the space we gain. There is one fact recalled to my mind by this case, which is curious and worthy of notice, namely, that the teeth in such cases although never used in mastication, are almost always found to be carious.

At future meetings the remaining steps of the operation will be successively exhibited.

The next case to which the Professor directed attention, was one in which a chancrous affection of the glans penis had resulted in destruction of the glans, and the anterior extremity of the corpus cavernosum, and in the obliteration of the anterior portion of the urethra, with a phymosis-like contraction of the integuments around the end of the stump.

In 19 out of 20 cases of obliteration of the urethra, with the formation of false passages for the urine, ulceration of the urethra takes place behind the scrotum, and the consequent infiltration of urine causes a fistulous opening in some portion of the perineum by which the fluid escapes. In the present case, on the contrary, this ulceration has occurred anteriorly to the perineum, and the urine has found its way into the corpus cavernosum, the cells of which are filled by it like a sponge, and the organ, from this cause, and from the inflammatory hardening of the parts, is maintained as it were in a constant state of erection, having more the appearance of that of one of our large domestic animals than that of a man. The injection of the urine into the corpus cavernosum in attempts to evacuate the bladder, has led to the establishment of these fistulous ulcers leading from the cells outwards on the body of the penis, through which, as exhibited by the lecturer, the urine can be at any time forced in small jets by pressing upon the distended body of the organ.

What is most astonishing in this case, is that this condition has been maintained for a space of two or three years without the occurrence of gangrene or sloughing of the parts: for one might have supposed it would have been attended with such consequences, or even have caused the death of the patient, for the rapid injection of urine into a highly organized part, is, from the irritating properties of that fluid, in common quickly followed by such disastrous results.

The plan by which I sometime since proposed to relieve this man, and which possibly will be adopted if he should make up his mind to bear the operation, was by a button hole incision into the posterior part of the urethra, to divert the flow of urine entirely from the corpus cavernosum; through which incision, after the fistulous openings in the body of the organ had closed, a trochar might be introduced and pushed out to the end of the penis, making a new urethra into which could be inserted a silver catheter in order to prevent the collapse of the new opening before its walls should have acquired the necessary firmness.

This, as remarked by the lecturer, is altogether a very singular case, and without counterpart in the annals of surgery; both from the anomalous course pursued by the urine, the position of the fistulous sinuses, and particularly from the impunity with which the vascular structure of the cavernous body has borne the presence of the irritating urine for so long a time.

The third case I present is one (a deep-seated oblong tumour in the perineum) which at first sight appears to be somewhat obscure.

But when we reflect upon the rarity of tumours occupying the anterior part of the perineum, except from one cause, namely, obstruction to the natural course or flow of the urine, (and we find that this patient has suffered for a long time from stricture of the urethra,) much of the obscurity will be dissipated. It is true that a common phlegmonous tumour, or enlarged muciparous glands, may appear in this situation, or there may be a hardened tumour of the parts, extending forwards, as the consequence of a stercoral abscess by the side of the rectum. But a careful inspection of the parts, with a close inquiry into the history of the case, will often serve to show the connection of these deep seated tumours in the front portion of the perineum, with a pre-existing lesion of the urethral passage—consisting in some form of stricture, injuries received upon the parts, or even from rough catheterism of the passage—indicating their dependence upon the escape of a small amount of urine into the cellular tissue between the inferior and middle fasciæ of the region.

Here we have a tumour of considerable size, deeply seated, and firm to the touch, occupying a great portion of the left side of the perineum. The history of its appearance is as follows. Some time since the patient had a gonorrhœa, which was *cured*, as he says, by balsam, injections, and the subsequent use of bougies. After the suspension of the treatment, he experienced difficulty in micturition, the urine dribbling away slowly, instead of flowing as usual in a continous stream. This difficulty increasing upon him, he soon after perceived this tumour commencing, and noticed also a slight burning pain after urination, beneath the commencing tumour, which has rapidly increased to its present condition.

We have here then, evidently stricture of the urethra, which too frequently follows such cases of gonorrhœa, and it is worth your while to notice this dribbling of the urine,—the falling of it, guttatim, as it were, from the end of the penis, as from this we are ju tified in suspecting the existence of more than one stricture, as I have observed that where but a single one existed, although it may be a narrow one, the urine issues in a continuous stream, and is thrown to some distance, which cannot be the case, where two or more are present, as the force of the current must then necessarily be broken.

By the introduction of a bougie, we find the canal obstructed by a firm stricture just anterior to the bulb, the instrument from its small size, having passed one which is seated nearer to the glans. The first and greatest is however, probably the cause of the tumour, which consists, as I have no doubt we shall find, of an infiltration of urine into the cellular structure, occurring anterior to the triangular ligament, and causing a local point of suppuration there, with the effusion of lymph around it so as to form a hardened base. Before we can expect a radical cure of the case, these strictures must be overcome, either by dilatation or division; but by attempting this in the first place, without giving any attention to the tumour we should subject the patient to the risk of an extensive and deep seated perineal abscess; I shall therefore do what I advise all of you, in such cases, never to hesitate in doing, namely, lay open fully and freely the whole tumour, and discharge its contents, allowing the urine, if it still escapes from the opening in the urethra, a free exit, and converting the affection into a fistula in perineo. This is best accomplished by the use of a sharp pointed bistoury which should be introduced at the posterior extremity of the tumour, in a position at right angles to the surface—and when carried into the cavity, which may be known by the freedom of motion in the point,—the handle should be depressed so as to bring the point out at the adverse boundary of the tumour, when by depressing the handle and cutting outwards the whole tumour is laid open, discharging, as you see, a quantity of urine with pus and blood. By this procedure we have removed the contents of the abscess and prevented its extension, and removed also the compression which it made upon the urethra, which otherwise would have rendered the use of bougies in dilating the strictures extremely difficult, and probably in the previous state of the parts more injurious than useful. As the patient presents the appearance of one with a broken constitution, which condition occurs so commonly in these disorders of the genital organs, he has been placed upon a tonic treatment, consisting of an infusion of uva ursi, buchu leaves and juniper berries with bicarb. of soda, which will be continued. The wound for the present will be simply dressed, and after the oozing of blood from the sides of the incision shall have ceased, be covered by a warm poultice.

Here is the case of a man begging lustily to have his leg cut off, in consequence of caries of the posterior part of the os calcis. This is a favor which, however, we never grant merely at the patient's request, nor ever resort to except in case of great necessity, knowing that it is but a very bad natural leg that is not better than a wooden one. Instead of it, therefore, we shall resort to the less serious operation of modern surgery, that of the excision of the carious bone. This patient has been for some time confined to his bed by this disease of the bone which has led to the establishment of a sinuous ulcer of the heel, from the margins of which these fungous excrescences are growing. This bone by the way, is much exposed to injuries and is peculiarly liable to become carious at its posterior part. Now to remedy this it will be necessary to lay the patient upon his abdomen, lay open the parts freely and remove the cause of ulceration whether it may be necrosed or carious bone, by means of a saw, rasparatory, or trephine. There is one objection to these operations upon the heel, which however cannot be avoided; that is, the fact that cicatrices in this part are almost always painful.

We will now commence, (having a tourniquet at hand in case of hemorrhage) by a semi-circular incision around the back part of the heel, then extending a straight one from the middle of this, through the ulcer, converting the wound into a kind of T shape, whereby plenty of space is gained and the external and internal plantar arteries avoided; upon raising the flaps from the bone, the first thing observed is a piece of dead bone lying partly loose upon the carious surface of the os calcis, after removing which and scraping away the carious surface, which is so soft as to be cut with the knife, we examine carefully with a probe, to see that no diseased portion is left behind; finding a portion still soft, we with the point of a rasparatory proceed to remove it, and when all is got rid of, we see a considerable depression is left in the surface of the bone. We next bring the flaps back to their places, with the intervention, however, of a piece of charpie, and over the whole apply a piece of lint spread with cerate,—confine it by a bandage and place the patient in bed, with the leg elevated on a pillow.

The next case to which I will ask your attention, is one of the caries of both phalanges of the great toe with a destruction of the intermediate joint; for which there is no earthly remedy except an amputation at the metatarso-phalangeal joint. Here you see two carious ulcers corresponding with the two bones of the toe, every effort to heal which, and the trial has been a long time making, has proved unavailing. Now there is a very serious objection to removing the great toe, which should always be considered before the operation is undertaken; and that is, by taking it away you destroy a part of the abutment upon which the arch of the foot rests, and therefore create some inconvenience. Many operations have been proposed and used in these amputations; for instance, *Graife*, when the disease did not involve the upper joint, employed the antique method of striking off the toe with a chisel and mallet. This cruel mode I need hardly say, is not used in our day; some recommend us to cut obliquely through the metatarsal bone, in order to avoid an alleged liability to abrasion by preventing the head of the bone, covered only by thin integument, from pressing against the inside of the boot; but if there is any real advantage gained in this point of view, it is more than counterbalanced by the removal of an unnecessary portion of the support required by the inside of the foot. The circular operation is also sometimes used, but that which I greatly prefer to any other is the oval operation of Scontetten. To perform this you commence your incision on the dorsum of the foot a little above the joint, bringing it down between the toes, around, under the bottom aud back to the starting point, making a kind of V shaped wound, with the ends of the V joined by a segment of a circle. An important modification, which is rarely noticed in the books, consists in so shaping your incision as to leave more flap upon the margin of the foot than upon the other where the commissure of the toes prevents retraction to any great extent.

### OPERATION.

You first bend the toe down and make your V incision, cutting to the bones. Then sever the tendons on the back of the joints; afterwards divide the ligaments, sweep your knife under the bone, and the toe is separated. You will generally find but one artery to tie as in the present instance, namely the continuation of the internal plantar. This done you proceed to bring the integuments together by your straps, and if the operation has been properly performed, there will appear to be a superabundance of tissue as you may see; this however is rather apparent than real, as your patient could meet with no greater misfortune than to have the integuments too tight and adherent to the end of the stump, as it would subject him to constant pain and ulceration in the parts. You will often find, too, a portion of the long flexor tendon projecting after the toe has been removed ; this should be cut, as tendons give no pain on division except when accompanied by a nerve, as was proved by Mr. Pott upon his own person, and moreover is peculiarly liable if exposed, to slough and give trouble. After well cleansidg and drying the parts, you bring them together by straps, over which a piece of lint cut so as to adapt itself to the stump, is confined by a tight roller bandage, applied so as to exercise some compression, which is well calculated to lessen the tendency to inflammatory action, and therefore very important in the treatment.

